# Gene polymorphisms and chronic obstructive pulmonary disease

**DOI:** 10.1111/jcmm.12159

**Published:** 2013-11-21

**Authors:** Xiaodan Wu, Bowei Yuan, Elena López, Chunxue Bai, Xiangdong Wang

**Affiliations:** a Department of Respiratory Medicine Zhongshan Hospital, Fudan UniversityShanghai, China; b Shanghai Respiratory Research InstituteShanghai, China; c Harbin Medical UniversityHarbin, China; d Department of Oncohematology of Children, Hospital Universitario Niño JesúsMadrid, Spain

**Keywords:** chronic lung diseases, COPD, gene, biomarkers, polymorphism, therapy

## Abstract

The genetic component was suggested to contribute to the development of chronic obstructive pulmonary disease (COPD), a major and growing public health burden. The present review aims to characterize the evidence that gene polymorphisms contribute to the aetiology of COPD and related traits, and explore the potential relationship between certain gene polymorphisms and COPD susceptibility, severity, lung function, phenotypes, or drug effects, even though limited results from related studies lacked consistency. Most of these studies were association studies, rather than confirmatory studies. More large-sized and strictly controlled studies are needed to prove the relationship between gene polymorphisms and the reviewed traits. More importantly, prospective confirmatory studies beyond initial association studies will be necessary to evaluate true relationships between gene polymorphisms and COPD and help individualized treatment for patients with COPD.

- Introduction
- Gene polymorphisms and COPD susceptibility
  Alpha 1-antitrypsin gene
  Tumour necrosis factor genes
  Microsomal epoxide hydrolase gene
  Glutathione S-transferases genes
  Transforming growth factor-beta(1) gene
  Other genes
- Gene polymorphisms and disease severity
- Gene polymorphisms and extent of emphysema
- Gene polymorphisms and exacerbations of COPD
- Gene polymorphisms and lung function decline
- Gene polymorphisms and COPD-related phenotypes
- Gene polymorphisms and COPD-related cardiovascular changes
- Gene polymorphisms and drug effects
- Perspectives

## Introduction

Chronic obstructive pulmonary disease is characterized by the development of airflow limitation that is progressive and not fully reversible 1, and is a major and growing public health burden as the fourth leading cause of death in the world according to 2002 statistics 2. Chronic obstructive pulmonary disease is expected to be one of the top five chronic diseases in terms of global mortality and morbidity by 2030 3. Chronic obstructive pulmonary disease was ranked as the fourth leading cause of death in urban areas and the third in rural areas in China by 2008 4. Cigarette smoking has been proposed as the most important environmental risk factor in the development of COPD, while only a minority of smokers develop clinically symptomatic COPD 5,6. These observations, together with the familial aggregation of COPD 8, indicate that genetic components contribute to the development of COPD 9,10. Gene polymorphisms are the allelic variation or point mutations in the DNA, including single-nucleotide polymorphisms. A gene can be polymorphic if more than one allele occupies the gene locus within a population. Most studies on gene polymorphisms have been carried out in cancer including lung cancer 12, while little was understood on genetic components, which may mainly contribute to the development of COPD. The present review aims to characterize the evidence that gene polymorphisms contribute to the aetiology of COPD and related traits, and explore the potential relationship between certain gene polymorphisms and COPD susceptibility, severity, lung function, phenotypes or drug effects, even though limited results from related studies lacked consistency.

## Gene polymorphisms and COPD susceptibility

### Alpha 1-antitrypsin gene

The relationship between gene polymorphisms and COPD susceptibility has been paid special attention and was explored in a large number of studies on hundreds of genes, although the results varied between studies and populations of people, as listed in Table 1. The initially studied polymorphism related to COPD susceptibility was Z allele of alpha 1-antitrypsin (AAT) gene. The AAT coding gene SERPINA1 is highly polymorphic, with more than 125 single-nucleotide polymorphisms (SNPs) reported, its most common being the normal M allele and its subtypes, besides the deficient alleles protease inhibitor (PI) S (caused by a glutamate to valine mutation at position 264) and PI Z (caused by a glutamate to lysine mutation at position 342) 13. These alleles contribute to variant genotypes including MM, MS, SS, MZ, SZ and ZZ, resulting in different levels of AAT and COPD susceptibility. A number of studies on the relationship between those gene polymorphisms and COPD are summarized in Table 2. Severe AAT deficiency, usually caused by the presence of two copies of mutant Z allele, was suggested as a genetic risk factor in about 80% of patients with COPD between 30 and 40 years or younger 14. Protease inhibitor Z allele is uncommon in most populations and the intact physiological role of AAT gene is not clear. Other polymorphisms of PI were also studied, but results were not consistent. A meta-analysis including 16 case–control studies and cross-sectional studies demonstrated that patients with PI MZ had higher risk to suffer from COPD as compared with those with PI MM homozygotes 15, while another meta-analysis found that individuals with PI SZ heterozygotes had significantly higher risk for COPD 16. Another study on SERPINA1 alleles of M1, PI M2, PI M3, PI S and PI Z found no significant differences in allele frequencies between COPD patients and healthy controls 17. Further studies are needed with enough cases to confirm the role of those gene homozygotes in the risk of COPD.

**Table 1:** Gene polymorphisms studied for COPD susceptibility

Genes	Polymorphisms	Study type	Subjects	Risk of COPD	Refs
Alpha1-antitrypsin	Protease inhibitor (PI) MZ	Meta-analysis	2175 COPD cases and 3480 controls in 11 case–control studies, 10338 participants in 5 cross-sectional studies	Increased risk (PI MZ *versus* MM)	15
	PI SZ, MS and SS	Meta-analysis	2237 COPD cases and 3926 controls in 12 case–control studies, 10539 participants in 5 cross-sectional studies	Increased risk (PI SZ), no effect (PI MS), unsure (PI SS)	16
	PI M1, M2, M3, S and Z	Association study	100 COPD patients and 200 controls	No effect	17
Tumour necrosis factor	TNF-308	Meta-analysis	2380 COPD cases and 3738 controls	Increased risk (TNF-308 A) in Asian populations	21
	LtalphaNcoI^*^1/2	Association study	66 COPD cases, 23 participants with disseminated bronchiectasis, 45 participants with non-obstructive pulmonary disease and 98 controls	No effect	22
Microsomal epoxide hydrolase (EPHX1)	EPHX1-113, EPHX1-139	Meta-analysis	1847 COPD cases and 2455 controls	Increased risk (EPHX1-113 and -139)	27
	T113C and A139G	Association study and a meta-analysis	47060 participants in association study; 7489 COPD cases and 42970 controls in meta-analysis	No effect on COPD risk from association study, minor effect on increased risk from meta-analysis	28
Glutathione S-transferase mu 1 (GSTM1)	Null/plus	Association study	184 COPD cases and 212 controls	Increased risk (GSTM1-null)	29
	Null/plus	Association study	204 COPD cases and 208 controls	Increased risk (GSTM1-null)	32
	Null/plus	Association study	50 COPD cases and 50 controls	No effect	33
GST theta 1 (GSTT1)	Null/plus	Association study	204 COPD cases and 208 controls	No effect	32
	Null/plus	Association study	50 COPD cases and 50 controls	Increased risk (GSTT1-null)	33
GSTP1	Homozygous isoleucine 105 GSTP1	Association study	184 COPD cases and 212 controls	Increased risk when in combination with at least one mutant mEPHX exon-3 allele and GSTM1-null	29
	Ile105Val	Association study	89 COPD cases and 94 controls	No effect	34
Transforming growth factor-beta 1 (TGF-beta1)	3′UTR rs6957, C-509T rs1800469 and Leu10Pro rs1982073	Association study	1156 participants without COPD and 188 with COPD	Increased risk	37
	rs1800469 and rs1982073	Meta-analysis	1508 COPD cases and 2608 controls	No effect	38
	rs6957, rs1800469, rs2241712, and rs2241718	Association study	160 COPD cases and 177 controls	No effect	38
Beta(2)-adrenoceptor (ADRB2)	ADRB2-16, 27 and 164	Association study	65 COPD cases and 41 controls	Increased risk (Gly16)	39
	ADRB2-16 and 27	Association study	1090 participants including 39 COPD patients and 221 controls	Increased risk (Arg16 homozygotes, Arg16/Gln27 haplotype)	40
	+46 A/G and +79 C/G	Association study	106 COPD cases and 72 controls	Increased risk (+79 C/G)	41
Tissue inhibitor of metalloproteinase-2	+853 G/A, −418 G/C	Association study	106 COPD cases and 72 controls	Increased risk (+853 G/A)	42
Toll-like receptor-4	Asp299Gly	Association study	152 COPD cases and 444 controls	Decreased risk	43
Endothelial nitric oxide synthase gene	−786T/C, −922A/G, 4B/4A, and 894G/T	Association study	190 COPD cases and 134 controls	Increased risk (−786C, −922G, and 4A)	44
Interleukin 8 (IL8)	IL8-351	Association study	53 COPD cases and 122 controls	Increased risk	45
Type IV collagen alpha3 gene	451R allele	Association study	311 COPD cases and 386 controls	Increased risk	46
Interleukin 6 (IL6)	IL-6 −174, −572 and −597	Association study	191 COPD cases, 75 smokers and 296 controls	Decreased risk (−572C)	47
	IL6 −174G/C	Association study	389 cases of COPD and 420 controls	Increased risk	48
A Disintegrin and metalloprotease 33 (ADAM33)	Q-1, intronic; S1, Ile –> Val; S2, Gly –> Gly; V-1 intronic; V4, in 3′ untranslated region	Association study	287 COPD cases and 311 controls	Increased risk	49
	V4, T + 1, T2, T1, S2, S1, Q-1, and F + 1	Association study	312 COPD cases and 319 controls	Increased risk	50
	V4, T + 1, T2, T1, S2, S1, Q-1, and F + 1	Association study	240 COPD cases and 221 controls	Increased risk	51
Vitamin D-binding gene	rs7041 and rs4588	Association study	262 COPD cases and 152 controls	Increased risk (rs7041 T allele)	55
Manganese-superoxide dismutase (Mn-SOD)and catalase gene	Ala16Val of Mn-SOD, −262C>T of catalase gene	Association study	165 COPD cases and 165 controls	No effect	57
Superoxide dismutase-3 (SOD3)	rs8192287, rs8192288, and rs1799895	Association study	389 COPD cases and 472 controls	No effect	58
Surfactant protein B	rs1130866, rs2077079 and rs3024791	Association study	10,231 participants from the general population	No effect	59

**Table 2:** Studies on relationship between alpha 1-antitrypsin gene polymorphisms and COPD

Polymorphisms	Study type	Subjects	Populations	Significance	Refs
Protease inhibitor (PI) MZ	Meta-analysis	15993 participants for risk study, 10823 participants for FEV(1) study	Caucasian	PI MZ was associated with an increased risk of COPD than PI MM. There was no difference in mean FEV(1) between PI MM and PI MZ individuals	15
PI SZ, PI MS and PI SS	Meta-analysis	16702 participants	Caucasian	PI SZ genotype was a significant risk factor for COPD. Protease inhibitor MS genotype was not associated with COPD risk after correcting for smoking. There were not enough cases to summarize the risk of COPD in PI SS homozygotes	16
PI M1, M2, M3, S and Z	Association study	100 COPD patients and 200 controls	Tunisian	There were no significant differences in allele frequencies between COPD patients and controls. None of the polymorphisms was related to the emphysema type and FEV(1) annual decline	17

### Tumour necrosis factor genes

Tumour necrosis factor α (TNFα) was proposed to play a critical role in COPD pathogenesis, *e.g*. neutrophil release and activation 18. The genomic polymorphism resulting in substitution of the nucleotide adenine (A) for guanine (G) at position −308 was discovered within the TNFα locus in 1992 19. Presence of the A substitution was related to increased production of TNFα 20. A large number of studies about the association of TNF-308 polymorphisms and COPD have been performed and are listed in Table 3, where an inconsistent association between COPD susceptibility and gene polymorphisms was observed. A meta-analysis of 24 eligible studies published between 1966 and 2009 demonstrated a significant association between TNF-308 polymorphisms and an increased risk of COPD (OR = 1.335, for allele A carriers *versus* G/G; OR = 1.330, for allele A *versus* allele G) 21. The TNF-308 A allele was suggested by subgroup analysis as a risk factor for the development of COPD in Asian populations rather than in Caucasians. Values of TNF gene complex polymorphism (LtalphaNcoI*1/2) and TNF-308 were also validated among COPD, disseminated bronchiectasis, non-obstructive pulmonary diseases, and healthy controls in Caucasoid individuals 22. This small population association study found that the TNF gene complex for the considered polymorphisms did not seem to be a major genetic risk factor in COPD.

**Table 3:** Studies on relationship between tumour necrosis factor (TNF) gene polymorphisms and COPD

Polymorphisms	Study type	Subjects	Populations	Significance	Refs
TNF-308	Meta-analysis	2380 COPD cases and 3738 controls	Asian and Caucasian	TNF-308 A allele might be a risk factor for developing COPD among Asian populations, but not among Caucasians	21
TNF gene complex polymorphism (LtalphaNcoI^*^1/2)	Association study	66 COPD cases, 23 participants with disseminated bronchiectasis, 45 participants with non-obstructive pulmonary disease and 98 controls	Caucasian	TNF gene complex polymorphism did not seem to play a major role as genetic risk factor in COPD	22
TNF-308	Association study	84 COPD patients	Japanese	TNF-308 A allele might be partly associated with the extent of emphysematous changes in patients with COPD	63
TNF-308	Association study	106 COPD patients and 99 controls	Caucasian	There was no increased frequency of the A allele in patients compared to control participants. AA homozygous patients had less reversible airflow obstruction and a significantly greater mortality	60

Interestingly, the association of TNF-308 polymorphism with asthma has only been reported in the U.K./Irish population 23 and in a European-American population 24, making it probable that this is not a polymorphism that confers risks of COPD among Asians only. Large populations of genome-wide association studies, especially prospective confirmation studies, are needed to confirm the association between TNF polymorphisms and COPD susceptibility and to interpret non-consistent findings among populations.

### Microsomal epoxide hydrolase gene

Microsomal epoxide hydrolase (EPHX1) plays an important role in the metabolism of highly reactive epoxide intermediates formed in cigarette smoke. The population was classified into fast, normal, slow and very slow groups of EPHX1 phenotypes, based on reported polymorphisms within the coding region of EPHX1 genes tyrosine/histidine 113 or histidine/arginine 139 25–26. The association between the genotypes and phenotypes of EPHX1 and COPD susceptibility varied among different populations. A meta-analysis of 16 eligible studies showed that the EPHX1 113 mutant homozygote was significantly associated with an increased risk of COPD 27. Similar to TNF-308 polymorphisms, more obvious association of EPHX1 139 heterozygote with the development of COPD was noted in the Asian population. The slow activity phenotype of EPHX1 was associated with an increased risk of COPD (OR: 1.67), while the fast activity phenotype of EPHX1 appeared more protective against the development of COPD in the Asian population. The very slow activity phenotype of EPHX1 was a risk for COPD development in the Caucasian population, rather than in the Asian population. However, an association study on Danish individuals did not find the association among patients with COPD diagnosed by spirometry, the EPHX1 genotypes or phenotypes, or in smokers or non-smokers, respectively 28. The meta-analysis of 19 studies on COPD patients and healthy controls showed limited variation among T113C heterozygotes and homozygotes or A139G to define the risk of the disease 28.

### Glutathione S-transferases genes

Glutathione S-transferases (GSTs) are key players to detoxify various aromatic hydrocarbons found in cigarette smoke. The proportion of GST mu 1 (GSTM1)-null genotypes was significantly higher in patients with COPD than in controls in a Taiwanese population 29, while there was no difference in the frequency of polymorphic genotypes of GST theta 1 (GSTT1) and GST P1 (GSTP1). One active allele in GSTM1 was found to have a protective effect against the development of COPD in patients with non-small-cell lung cancer 30. A study conducted in Dubai demonstrated that carriers with null GSTM1 genotype had a high risk of developing COPD, especially with both null GSTT1 and GSTM1 haplotype 31. The frequency of homozygous GSTM1 null genotype was significantly higher in Indian patients with COPD 32, while there was no significant difference in the distribution of homozygous null GSTT1. However, those results were not confirmed in another Indian study, where GSTT1 null genotypes rather than GSTM1 null genotypes were associated with the susceptibility to COPD despite the relatively small sample size 33. The Ile105Val polymorphism of GSTP1 was not associated with development of COPD in Koreans 34. The Taiwanese study found that the combination of genetic variants, *e.g*. one mutant mEPHX exon-3 allele, GSTM1-null and homozygous isoleucine 105 GSTP1 genotypes, could be valuable indicators of susceptibility to COPD 29. However, there was no association of GSTT1, GSTM1 and GSTP1 polymorphisms with COPD in the Chinese population of Hongkong and Southern China 35.

### Transforming growth factor-beta(1) gene

Abnormalities in the TGF-β1 gene were found to be associated with COPD susceptibility. Two SNPs (rs2241712 and rs1800469) in the promoter region of TGF-β1 and one SNP (rs1982073) in exon 1 of TGF-β1 were discovered to be significantly associated with COPD 36. A significantly higher prevalence of carriers of the minor allele of TGF-β1 rs6957 SNP was found in patients with COPD compared with the general population 37. No association between four SNPs (rs6957, rs1800469, rs2241712, and rs2241718) of TGF-β1 and increased risks of COPD was found in genotyped COPD cases and control subjects 38. No correlation between increased risks of COPD in carriers of the T allele (TT+TC) and the CC genotype in rs1800469 and rs1982073 was observed in a meta-analysis 38. Increased risks with the rs1800469 T allele were identified only in Caucasian subjects (OR: 1.53) and not in Asians.

### Other genes

Numerous other gene polymorphisms are also studied with relation to COPD susceptibility. The Gly16 polymorphism of the beta(2)-adrenoceptor (ADRB2) gene might be associated with the susceptibility to the development of COPD in a Chinese population 39. The Arg16 homozygotes of ADRB2 gene was found to be associated with an increased risk of COPD (OR: 5.13) in Caucasian participants 40. Besides, the Arg16/Gln27 haplotype was associated with COPD (OR: 2.91). The distribution of the genotype frequencies of ADRB2+79 C/G was significantly different between COPD and control groups in an Egyptian population, suggesting a role of +79 C/G in pathogenesis of COPD 41. Two polymorphisms of the tissue inhibitor of metalloproteinase2 gene, +853 G/A and −418 G/C, were found to be associated with the development of COPD in the Japanese population, while +853 G/A rather than −418 G/C in the Egyptian population 42. The frequency of the Asp299Gly polymorphism of the Toll-like receptor-4 was observed to be significantly decreased in COPD patients 43. The endothelial nitric oxide synthase gene, −786C, −922G, and 4A alleles, associated haplotypes and genotype combinations, were found to be overrepresented in COPD patients 44. The polymorphisms at interleukin 8 (IL-8) −351 45, type IV collagen alpha3 gene 46 were associated with an increased risk of COPD. Interleukin-6 −572C allele was suggested to confer a diminished risk of developing COPD in a Spanish population 47, while IL6-174G/C SNP increased risk of COPD in Canadian smokers 48. Five SNPs in A Disintegrin and metalloprotease 33 (ADAM33) were associated with Caucasian COPD 49. Association between ADAM33 polymorphisms and COPD risk was also found in northeastern China 50 and Tibet 51. Iron regulatory protein 2 SNPs 52, macrophage scavenger receptor-1 gene coding SNP P275A 53 and Hedgehog-interacting protein gene SNPs 54 were demonstrated to be associated with COPD susceptibility. Homozygous carriers of the rs7041 T allele in the vitamin D-binding gene were found to be associated with increased risks for COPD (OR: 2.11) 55.

Some studies failed to find an association between certain polymorphisms and COPD risk. Neither IL-1beta polymorphisms at position −511 base and at the amino acid residue 105 nor IL-1 receptor antagonist polymorphisms in intron 2 were associated with susceptibility to Japanese with COPD 56. No difference of tandem repeat in IL-4 as well as +1111 C/T and +2044 G/A in IL-13 was noted between Japanese patients with COPD and controls or between Egyptian subjects 41. There were no significant differences in the distribution of the different genotypes or allele frequencies between patients and controls for both the manganese-superoxide dismutase gene and catalase genes 57. Three superoxide dismutase-3 polymorphisms were not related to COPD susceptibility 58. In a Danish population, the functional polymorphisms in the surfactant protein B gene were not associated with risk of COPD 59.

Genes might be related to each other functionally or connected in certain pathways or networks; however; little was known about the combined effects of two or more gene polymorphisms on COPD susceptibility, which may be a developing direction for future study.

Although several studies including some large-population and well-performed studies have demonstrated the relationship between certain gene polymorphisms and COPD susceptibility, there have been little prospective confirmatory studies to further confirm whether certain polymorphisms could truly increase or decrease COPD risk. Although it will take many years for patients to develop COPD and make follow-up work much tougher, prospective studies are more meaningful and might point to a new direction for future study.

## Gene polymorphisms and disease severity

Spirometric assessment based on airflow limitation is one of the most important components to evaluate the severity of COPD, *e.g*. patients with mild COPD have the forced expiratory volume in one-second (FEV1) of ≥80% of the predicted value, moderate with 50%≤FEV1<80% predicted, severe with 30%≤FEV1<50% predicted, or very severe patients have FEV1<30% predicted. Chronic obstructive pulmonary disease patients with AA homozygous at position −308 on the TNFα gene had less reversible airflow obstruction and a significantly higher mortality on a 2-year follow-up 60. There was a significant correlation between the Gln27 ADRB2 polymorphism and FEV(1) percent predicted value in the Chinese population 39, between Arg16 homozygotes of ADRB2 and an increased risk of symptoms of wheeze in Caucasian participants 40, or between the homozygous variant of EPHX exon 3, the GSTM1-null genotype and independent risk factors for developing severe COPD in the Taiwanese population 29. A family-based study demonstrated that a SNP in the promoter region of TGF-β1 (rs2241712) and two SNPs in the 3′ genomic region of TGF-β1 (rs2241718 and rs6957) were significantly associated with the alterations of FEV1 in pre- and post- application of bronchodilator 36. VV470 genotype of cystic fibrosis transmembrane conductance regulator gene was suggested to be associated with mild/moderate COPD (<50% pred) in a Serbian population 61. Four SNPs (Q-1, S1, S2 and V-1) in ADAM33 were associated with lung function abnormalities in 880 Caucasians 49. Study on 26 SNPs in matrix metalloproteinase- 1, 9 and 12 genotyped from 977 COPD patients and 876 non-diseased smokers of European descent implicated haplotypes of MMP-12 as modifiers of disease severity 62. The macrophage scavenger receptor-1-coding SNP P275A was associated with poorer measures of lung function 53, and GSTM1 null polymorphism with pulmonary function in a north Indian population with or without smoking 32.

On the other hand, some studies failed to find an association between polymorphisms and COPD severity. Some meta-analysis-based studies demonstrated that there was no difference of mean FEV1 between PI MM and PI MZ individuals 15, and between PI MS and PI MM individuals 16. Danish studies found that there was no significant association between the functional polymorphisms of the SP-B gene (rs1130866, rs2077079 and rs3024791) and lung function or hospital admissions of COPD 59, or between COPD hospitalization and EPHX1 genotypes or phenotypes in smokers or nonsmokers, respectively 28.

## Gene polymorphisms and extent of emphysema

A visual scoring system was used to evaluate the ratio of the low attenuation area to the corresponding lung area in patients with COPD (*n* = 84) 63, where TNFα-308*1/2 allele frequency differed between patients with a visual score <11 and ≥11. It indicates that the TNF-alpha-308 polymorphism may be, at least partly, associated with the extent of emphysematous changes in patients with COPD. Three SNPs of CC chemokine ligand 5 (CCL5) gene (−403G>A, −28C>G and 375T>C) were genotyped in COPD patients (*n* = 267) and the −28G allele was inversely associated with the CT score 64. Functional single-nucleotide polymorphisms in the CCL5 gene were associated with milder emphysema. Three superoxide dismutase-3 polymorphisms were genotyped in severe COPD cases from the National Emphysema Treatment Trial (NETT, *n* = 389) and smoking controls from the Normative Aging Study (NAS, *n* = 472) 58. The minor alleles of SNPs E1 and I1 were associated with a higher percentage of emphysema diagnosed by chest computed tomography. Besides, the association with E1 was replicated in a family study, where the minor allele was associated with more emphysema.

## Gene polymorphisms and exacerbations of COPD

Mannose-binding lectin (MBL) is a pattern-recognition receptor in serum that assists innate immunity by binding to exposed sugars on the surface of invading bacteria 65–66. Polymorphisms of the MBL2 gene reduced serum MBL levels and were associated with risk of infection 67. Australian researchers found that the MBL2 codon 54 B allele affected serum levels of MBL in patients with COPD infective exacerbation, as compared with smokers with normal lung function, while patients carrying the low MBL-producing B allele had high risk of admission for infective exacerbation 68. A prospective study among 215 patients with COPD included 96 recurrent exacerbators with three or more episodes of infective exacerbation over 3 years and 119 less-frequent exacerbators with two or fewer episodes or 137 healthy individuals in Taiwan demonstrated that 12 among the recurrent exacerbators had the MBL deficiency genotype compared with five among the less-frequent exacerbators 69. In addition, the frequency of infective exacerbation was significantly higher in patients with MBL-deficient genotypes than those with non-MBL-deficient genotypes. It seems that MBL 2 deficiency because of MBL2 polymorphisms might increase the risk of recurrent infective exacerbation in COPD patients.

## Gene polymorphisms and lung function decline

Clinical studies demonstrated that the distribution of the IL-1beta and IL-1 receptor antagonist gene haplotypes was different between Canadian smokers with a rapid decline in lung function and smokers with no decline in lung function, although the genotypes were not associated with the rate of decline of lung function 70. Another Canadian study found that three of seven IL6 SNPs were associated with decline in FEV 1, of which the IL6 -174C allele was associated with a rapid decline in lung function 46. A total of 21 SNPs of leptin receptor (LEPR) gene were significantly associated with lung function decline over a 5-year follow-up period in a population of 429 European-Americans 71. The individual allele of SNP5G, SNP6A, SNP7G and SNP8T were associated with rapid decline in FEV1 despite smoking cessation, when 82 COPD patients (ex-smokers) were prospectively followed up for 30 months and evaluated the differences among the genotypes in the annual rate of decline in FEV1 with ten SNPs in and around the cell division cycle 6 (CDC6) gene 72. The longitudinal effect on lung function of two endothelin-1 gene polymorphisms (+138insA/delA and Lys198Asn) was analysed in a population of 190 smokers with or without COPD 73. The adjusted annual decline of FEV1 was greater for those having at least one copy of the mutated gene ins/delA compared with those with the wild-type allele both in the non-COPD smokers group and in COPD smokers. On the contrary, those heterozygous for the Lys198Asn polymorphism were found to have a slower decline in FEV1 compared with those homozygous for the wild-type allele.

Some gene polymorphisms were indicated not to be related to lung function decline. For example, Decorin (an extracellular matrix proteoglycan) SNPs, TGF-β1 SNPs and their haplotypes were not associated with accelerated FEV1 decline in 1390 cases in the Netherlands 37. Eleven SNPs of the genes encoding IL-10 and the alpha subunit of its receptor (IL10RA) were not associated with the rate of decline in FEV1 in smoking-induced COPD 74. Carriers of tested polymorphisms (PIM1, PIM2, PIM3, PIS and PIZ) of Alpha-1 antitrypsin gene were not associated with the annual decline of FEV1 determined for 2 years in a Tunisian study 17.

## Gene polymorphisms and COPD-related phenotypes

Gene polymorphisms of hematopoietic cell kinase (Hck) were found to be associated with COPD-related phenotypes in a Canadian population 75. The 15 bp insertion/deletion polymorphism 8656 L/S polymorphism was associated with smoking on baseline lung function and bronchodilator response, and with the expression of Hck protein and polymorphonuclear leucocyte myeloperoxidase release. The association of eight SNPs of TGF-β1 with the emphysema phenotype was investigated in a Japanese population of 70 COPD patients with emphysema phenotype and 99 healthy smokers 76. The frequency of one significant haplotype structured by the eight SNPs was significantly higher in the group with emphysema than in the healthy smokers. In addition, rs1800469T and rs1982073C alleles were significantly associated with severe airflow limitation. The 4A and T alleles of endothelin-1 polymorphisms were found to be associated with emphysematous and bronchitic phenotypes for patients with COPD 77. It was also proposed that disease subtypes and/or related phenotypic variables even in a highly selected group of severe emphysema patients were associated with TGFβ1 SNP rs1800470 78.

There are also negative results concerning the relationship between gene polymorphisms and COPD-related phenotypes. For example, a cross-sectional study revealed no significant relationship between common SERPINA1 polymorphisms (PIM1, PIM2, PIM3) and the emphysematous type of COPD 17.

## Gene polymorphisms and COPD-related cardiovascular changes

Whether polymorphisms of the renin angiotensin system, *e.g*. angiotensinogen (M235T), angiotensin-converting enzyme (I/D), and angiotensin II type 1 receptor (A1166C) were associated with right ventricular hypertrophy diagnosed by electrocardiography was investigated in 87 patients with severe COPD 79. The angiotensin-converting enzyme DD genotype was negatively associated with right ventricular hypertrophy in male patients, rather than in female patients. Polymorphisms of angiotensinogen and angiotensin II type 1 receptor genes were not associated with right ventricular hypertrophy. The association of deletion (D)/insertion (I) polymorphism in angiotensin-converting enzyme gene with pulmonary hypertension was determined in 19 patients with COPD with right heart catheterization followed by a constant-load exercise test 80. The pulmonary arterial pressure (Ppa) and pulmonary vascular resistance (Rpv) in patients with the DD genotype after exercise challenge was significantly higher than in patients with the genotype II. Although it remains the first study to investigate the relationship between gene polymorphisims and pulmonary hypertension evoked by exercise challenge, the results need further validation because of the small sample size. Polymorphisms of angiotensin-converting enzyme and endothelial nitric oxide synthase genes polymorphisms were associated with pulmonary hypertension in patients with COPD 81. Mean values of Ppa in patients with the BB genotype of synthase gene were significantly higher than in those with the non-BB genotypes. Polymorphisms of the cytokines *e.g*. IL-6, monocyte chemoattractant protein-1, and IL-1β, were hypothesized to be associated with the risk for pulmonary hypertension in COPD 82. The mean value of pulmonary artery pressure in patients with IL-6 GG genotype was significantly higher than in those with IL-6 CG or CC, when comparing plasma levels of cytokines and the polymorphisms G(−174)C of IL-6, C(−511)T of IL-1beta, and A(−2518)G of MCP-1 in 148 COPD patients with right heart catheterization data and 180 control participants.

## Gene polymorphisms and drug effects

A randomized, double-blind and crossover study enrolling 36 COPD patients found that the mean pulmonary arterial pressure, pulmonary vascular resistance (PVR), and lactate concentration after exercise were lower in patients with II or ID genotypes of angiotensin-converting enzyme after the treatment with captopril, rather than those with the DD genotype 83. Values of mixed venous oxygen tension in patients with the II genotype and treated with captopril were higher after exercise, but not in those with other genotypes. Of ADRB2 gene polymorphisms (Arg16Gly and Gln27Glu), the Arg16 allele was associated with lower bronchodilating responses to beta2-agonist inhalation in patients with COPD 84. The Arg16-Gln27 haplotype was also significantly associated with decreased response to salbutamol. In a prospective study recruiting 87 smokers with COPD, patients with wild-type GG genotype of corticotrophin-releasing hormone receptor 1 (CRHR1) gene and treated with fluticasone propionate and salmeterol for 12 weeks had significantly higher change of FEV1 than in GT heterozygotes 85. Improved FEV1 following inhaled corticosteroid and a long-acting beta2-agonist was associated with CRHR1 genetic polymorphism in patients with COPD.

Certain gene polymorphisms relating to COPD susceptibility will lead to the development of individualized medicine. The AAT coding gene polymorphisms were used to diagnose patients with severe AAT deficiency, leading to the therapy different from other COPD patients. Gene polymorphisms have also been found to contribute to inter-individual differences in the response to cigarette smoke, which could lead to more targeted anti-smoking interventions. Gene polymorphisms might be used to predict disease severity, complications, prognosis and drug effects for COPD patients individually. Furthermore, it is also important to identify and validate gene polymorphisms-specific biomarkers to trace and define the biological effects of genetic factors. Clinical bioinformatics, as an emerging discipline 86–94, covering metabolic and signalling pathways, biomarker discovery and development, computational biology, omics technology, high-throughput image analysis, human molecular genetics, human tissue bank, network medicine and systems biology, may be helpful for identification and validation of those polymorphism-specific biomarkers.

## Perspectives

Numerous studies have shown that gene polymorphisms may contribute to COPD susceptibility, severity, extent of emphysema, acute exacerbations, lung function, phenotypes, cardiovascular changes and drug effects, even though results are still conflicting. Most of these studies were association studies, rather than confirmatory studies. More large-sized and strictly controlled studies are needed to prove the relationship between gene polymorphisms and the reviewed traits. More importantly, prospective confirmatory studies beyond initial association studies will be necessary to evaluate true relationships between gene polymorphisms and COPD. Those studies will help individualized treatment for COPD patients.
